# Glyceraldehyde 3-Phosphate Dehydrogenase on the Surface of *Candida albicans* and *Nakaseomyces glabratus* Cells—A Moonlighting Protein That Binds Human Vitronectin and Plasminogen and Can Adsorb to Pathogenic Fungal Cells via Major Adhesins Als3 and Epa6

**DOI:** 10.3390/ijms25021013

**Published:** 2024-01-13

**Authors:** Aneta Bednarek, Dorota Satala, Marcin Zawrotniak, Angela H. Nobbs, Maria Rapala-Kozik, Andrzej Kozik

**Affiliations:** 1 Department of Analytical Biochemistry, Faculty of Biochemistry, Biophysics and Biotechnology, Jagiellonian University, Gronostajowa 7, 30-387 Kraków, Poland; aneta.bednarek@doctoral.uj.edu.pl; 2 Doctoral School of Exact and Natural Sciences, Faculty of Biochemistry, Biophysics and Biotechnology, Jagiellonian University, Gronostajowa 7, 30-387 Kraków, Poland; 3 Department of Comparative Biochemistry and Bioanalytics, Faculty of Biochemistry, Biophysics and Biotechnology, Jagiellonian University, Gronostajowa 7, 30-387 Kraków, Poland; dorota.satala@uj.edu.pl (D.S.); marcin.zawrotniak@uj.edu.pl (M.Z.); maria.rapala-kozik@uj.edu.pl (M.R.-K.); 4 Bristol Dental School Research Laboratories, University of Bristol, Dorothy Hodgkin Building, Whitson Street, Bristol BS1 3NY, UK; angela.nobbs@bristol.ac.uk

**Keywords:** *Candida albicans*, *Nakaseomyces glabratus*, moonlighting proteins, glyceraldehyde 3-phosphate dehydrogenase, vitronectin, plasminogen, agglutinin-like sequence protein 3, epithelial adhesin 6

## Abstract

*Candida albicans* and other closely related pathogenic yeast-like fungi carry on their surface numerous loosely adsorbed “moonlighting proteins”—proteins that play evolutionarily conserved intracellular functions but also appear on the cell surface and exhibit additional functions, e.g., contributing to attachment to host tissues. In the current work, we characterized this “moonlighting” role for glyceraldehyde 3-phosphate dehydrogenase (GAPDH, EC 1.2.1.12) of *C. albicans* and *Nakaseomyces glabratus*. GAPDH was directly visualized on the cell surface of both species and shown to play a significant part in the total capacity of fungal cells to bind two selected human host proteins—vitronectin and plasminogen. Using purified proteins, both host proteins were found to tightly interact with GAPDH, with dissociation constants in an order of 10^−8^ M, as determined by bio-layer interferometry and surface plasmon resonance measurements. It was also shown that exogenous GAPDH tightly adheres to the surface of candidal cells, suggesting that the cell surface location of this moonlighting protein may partly result from the readsorption of its soluble form, which may be present at an infection site (e.g., due to release from dying fungal cells). The major dedicated adhesins, covalently bound to the cell wall—agglutinin-like sequence protein 3 (Als3) and epithelial adhesin 6 (Epa6)—were suggested to serve as the docking platforms for GAPDH in *C. albicans* and *N. glabratus*, respectively.

## 1. Introduction

One of the main groups of microbial virulence factors includes proteins that are exposed on the pathogen cell surface and mediate contact and interactions with host tissues [1,2]. No exception to this, the major fungal pathogens for humans that, until recently, have been classified into a single genus, *Candida*, express several families of dedicated adhesins, covalently anchored to the glucan network of the cell wall [3]. The best characterized representatives are agglutinin-like sequence (Als) proteins in *C. albicans* [4]. However, the candidal cell wall also contains proteins that are more loosely adsorbed to the cell surface in a less predictable or poorly controlled manner [5,6,7]. Some of these match the criteria that Jeffery proposed in 1999 to define a special type of protein multifunctionality called “moonlighting” [8]. A moonlighting protein plays a well-established evolutionarily conserved function at an intracellular or extracellular location, as determined by the conventional mechanisms of protein sorting [9], but may also appear in atypical places and exhibit an additional function(s) [6,10,11,12,13]. An intracellular enzyme, glyceraldehyde 3-phosphate dehydrogenase (GAPDH, EC 1.2.1.12), which plays a highly conserved role in the glycolysis pathway by catalyzing the conversion of glyceraldehyde 3-phosphate into 1,3-diphosphoglycerate, is an example of a moonlighting protein. The occurrence of GAPDH on the cell surface and additional cell surface-dependent functions has been characterized, at least to some extent, for several bacterial pathogens [14,15,16,17,18,19,20,21], but only occasionally for pathogenic fungi. GAPDH was detected on *C. albicans* cells [22,23,24] and suggested to act as an adhesin, as deduced from the observed interactions with host proteins such as laminin and fibronectin [25]. GAPDH has also been proposed to contribute to the evasion of the host immune response, e.g., by binding to proteins that regulate the activity of the complement system [26]. Recently, GAPDH has been repeatedly found within cell wall preparations from *C. albicans* and a number of *Candida* non-albicans species using proteomics [27,28,29,30].

In the current work, we comparatively analyzed the moonlighting protein status of GAPDH in *C. albicans* and *Nakaseomyces glabratus* (formerly *C. glabrata*)—two species that both cause human candidiasis but differ to some extent regarding pathogenic mechanisms [31]. *C. albicans* is the most common candidal infectious agent; however, it is an opportunistic fungus and a natural component of the resident human microbiome residing in 30–70% of healthy individuals [32,33]. Its pathogenicity can manifest particularly in individuals with a weakened immune system, causing dangerous and difficult-to-treat infections [34,35,36]. *N. glabratus* has been much less studied as a fungal infectious agent, although in terms of infection rate it can be second after *C. albicans*, at least in some geographic regions [37,38]. Its virulence characteristics are presumed to have evolved independently of other *Candida* species, as it is phylogenetically closer to the non-pathogenic yeast *Saccharomyces cerevisiae* [39]. Unlike other common fungal pathogens, *N. glabratus* does not exhibit the ability to change morphological form from blastospore to hyphae. This species exhibits a high resistance to many antifungal drugs [40], making *N. glabratus*-derived candidiasis difficult to effectively treat, particularly in cases where the infection is severe or has spread to multiple parts of the body. Consequently, infections caused by this species have a high mortality rate among at-risk patients, making this species of particular interest and worthy of detailed study [41].

In this work, we provide direct evidence for the presence of GAPDH on the cell surface of *C. albicans* and *N. glabratus* and propose a significant role for this enzyme in contributing to the total fungal cell binding capacity to two selected host proteins—vitronectin (VTR) and plasminogen (HPG). Using purified fungal GAPDH, we quantitatively characterized its binding to VTR and HPG. Finally, we demonstrate that purified GAPDH can bind to whole fungal cells and this GAPDH adsorption is mediated by major dedicated adhesins. Such a mechanism may indicate how soluble GAPDH can be deposited onto the fungal cell surface at sites of fungal infection. 

## 2. Results

### 2.1. Occurrence of GAPDH on the Surface of C. albicans and N. glabratus Cells

**Figure 1:**
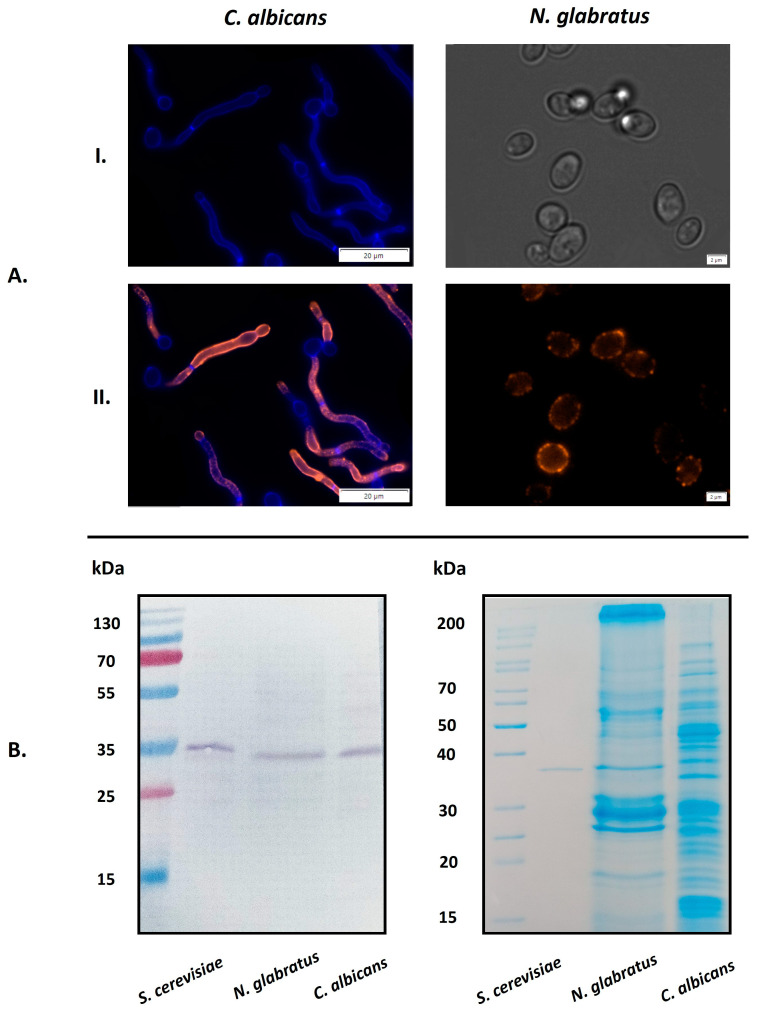
Visualization of GAPDH on *C. albicans* and *N. glabratus* cells by fluorescence microscopy. (**A**) *C. albicans* cells were incubated in RPMI 1640 medium at 37 °C for 3 h in the wells of a glass bottom 96-well microplate coated with poly-L-lysine (10^6^ cells per well). *N. glabratus* cells were grown in 20 mL of RPMI 1640 medium for 72 h in a glass flask and then transferred to 1.5 mL Eppendorf tubes (3 × 10^7^ cells per tube). After culturing, the presence of surface-exposed GAPDH was detected using a rabbit polyclonal anti-ScGAPDH antibody and mouse Alexa Fluor 488 dye-conjugated anti-rabbit secondary antibody. Before microscopic visualization, *N. glabratus* cells were transferred into the 96-well microplate (1**A**(**II**)). The photos were taken with an Olympus IX73 microscope. For controls, the cell wall of filamentous forms of *C. albicans* was stained with Calcofluor White dye (1**A**(**I**), left panel) and *N. glabratus* cells were observed under transmitted light (1**A**(**I**), right panel). (**B**) Western immunoblotting identification of GAPDH in cell-wall preparations from *N. glabratus* or *C. albicans* using an anti-ScGAPDH antibody (**left** panel). Purified ScGAPDH was used as a positive control. The full protein profiles for both candidal species were visualized by Coomassie Brilliant Blue G250 staining of SDS-PAGE gels (**right** panel). Scale bars: 20 µm for *C. albicans*, 2 µm for *N. glabratus*.

*C. albicans* and other related species present a specific protein profile on the cell surface that can vary according to external conditions [28,42]. The presence of GAPDH in the candidal “surfaceome” has been reported after culturing in different growth media [30,43]. Here, we directly visualized GAPDH on the surface of living intact cells of *C. albicans* and *N. glabratus* using specific antibodies directed against the *S. cerevisiae* enzyme (Figure 1A). For these experiments, we used *C. albicans* strain ATCC10231 and *N. glabratus* strain ATCC2001 grown in RPMI 1640 medium at 37 °C, reflecting to some extent conditions found at an infection site. Under these conditions, *C. albicans* forms invasive hyphae, while *N. glabratus* exists in yeast-like form. The cross-reactivity of anti-*S. cerevisiae* GAPDH antibody (anti-ScGAPDH) with *C. albicans* and *N. glabratus* enzymes (CaGAPDH and NgGAPDH, respectively) is substantiated by the known high-sequence similarity between candidal enzymes and baker’s yeast GAPDH (ScGAPDH)—78.2% and 87% amino acid identity, respectively (Expasy, SIB Swiss Institute of Bioinformatics) [44]. This was confirmed in this study using the purified candidal GAPDH preparations. Furthermore, using Western immunoblotting with the anti-ScGAPDH antibodies, GAPDH was detected in the mixtures of candidal cell wall proteins (CWP), isolated as described previously [5] with β-1,6-glucanase (Figure 1B). As seen in Figure 1B (left panel), the anti-ScGAPDH antibodies stained only a single band of entire CWP mixture, which was shown by mass spectrometric analysis to represent GAPDH (results not presented), thus confirming the specificity of used antibodies and additionally supporting the visualization results presented in Figure 1A.

### 2.2. Contribution of GAPDH to the Total Capacity of C. albicans and N. glabratus Whole Cells to Bind Human VTR and HPG 

**Figure 2:**
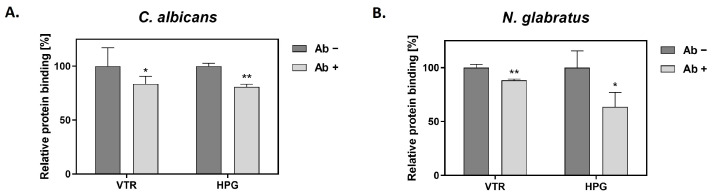
The effects of an anti-ScGAPDH antibody on the adsorption of VTR and HPG to the candidal cell surface. Binding experiments were performed for *C. albicans* (**A**) and *N. glabratus* (**B**) in MaxiSorp microplate wells (1 × 10^6^ cells per well incubated in RPMI 1640 medium for 3 h at 37 °C) or in Eppendorf tubes (3 × 10^7^ cells per tube), respectively. Cells were pre-incubated with the antibody (Ab) followed by the addition of biotin-labeled VTR and HPG, and final detection with a streptavidin-horseradish peroxidase/3,3′,5,5′-tetramethylbenzidine (SA-HRP/TMB) system. Statistical significance was analyzed with one-way ANOVA and marked as * for *p* < 0.05 or ** for *p* < 0.01.

Colonization of the human host by *C. albicans* involves the binding of candidal cells to the host extracellular matrix (ECM) and blood proteins [45]. *C. albicans* GAPDH (CaGAPDH) was previously identified within a set of proteins that interact with fibronectin, laminin, factor H and HPG [25,26,27]. What has never been estimated, however, is the relative contribution made by GAPDH to the binding of a given host protein by whole cells of *C. albicans* and related species. We addressed this problem with respect to two selected human proteins—VTR and HPG. VTR is a multifunctional adhesive glycoprotein that is a component of ECM and blood [46], whereas HPG is an inactive precursor of a potent serine protease (plasmin) and thus the essential component of the fibrinolytic system [47]. We analyzed the capacity for a specific anti-ScGAPDH antibody to block the recruitment of VTR or HPG to the surface of *C. albicans* and *N. glabratus* cells (Figure 2). For the filamentous form of *C. albicans*, binding in the presence of an antibody was decreased by 16% for VTR and 19% for HPG, while for *N. glabratus* blastospores, the reduction was 12% and 36%, respectively.

### 2.3. Purification and Basic Molecular Characterization of CaGAPDH and NgGAPDH

**Figure 3:**
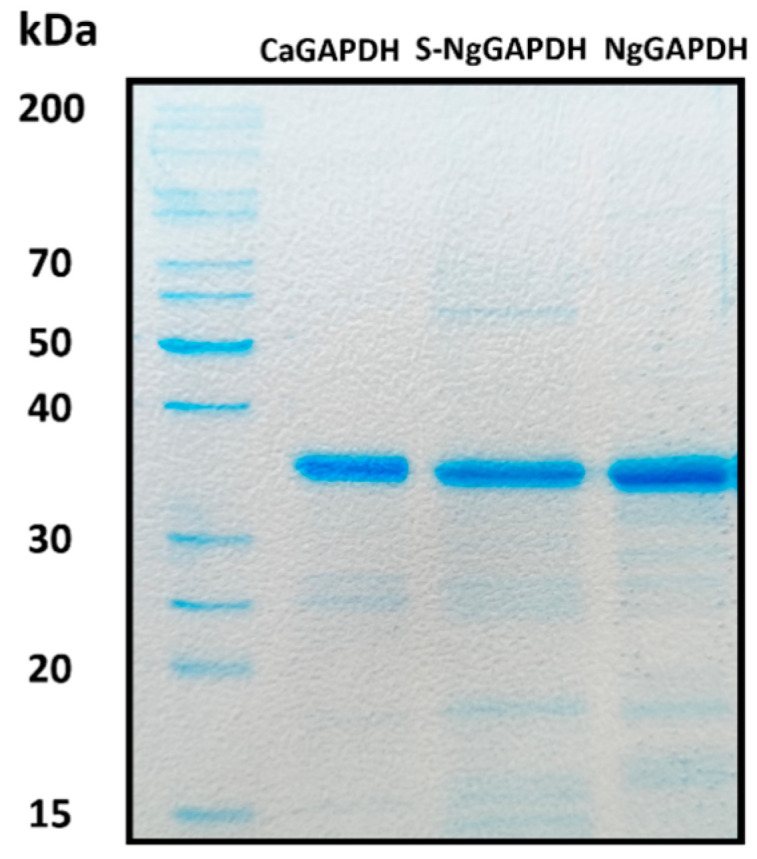
SDS-PAGE characteristics of isolated and purified forms of candidal GAPDH. Shown are recombinant CaGAPDH, S-NgGAPDH and cytoplasmic NgGAPDH. After SDS-PAGE, the gel was stained with Coomassie Brilliant Blue G250.

**Figure 4:**
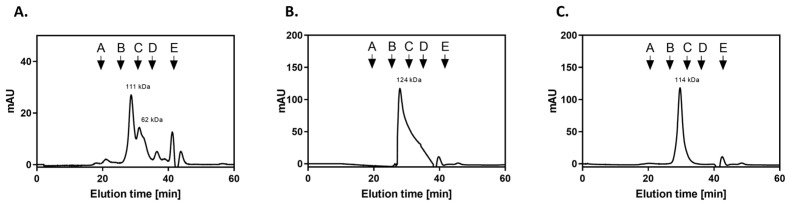
Characterization of candidal GAPDH preparations by gel filtration on Superdex 200 HR 10/50 column. The chromatography of recombinant CaGAPDH (**A**), NgGAPDH isolated from cytoplasm (**B**) and S-NgGAPDH (**C**) was performed in 50 mM NaH_2_PO_4_, 150 mM NaCl pH 7.0 buffer, with a flow rate of 0.5 mL/min. The Superdex 200 HR 10/50 column was calibrated with a molecular mass standard mixture containing thyroglobulin, 670 kDa (A); γ-globulin, 158 kDa (B); ovalbumin, 44 kDa (C); myoglobin, 17 kDa (D); and vitamin B12, 1.35 kDa (E).

For further planned experiments, preparations of purified GAPDH from two candidal species were required. A recombinant CaGAPDH was obtained by heterologous expression in *Escherichia coli*. The enzyme of *N. glabratus* was mostly used as a native protein preparation isolated from the cytoplasm, although a low-activity enzyme was also obtained from the cell wall (S-NgGAPDH). Representative purification steps for GAPDH from *N. glabratus* cytoplasm are shown in Table 1. The specific enzymatic activity of NgGAPDH was typically in an order of 6 U/mg—only two-fold lower than that of commercial ScGAPDH (ca. 12 U/mg) as measured with the same activity assay. The activity of recombinant CaGAPDH was significantly lower (<2 U/mg), but this was sufficient for further study. The basic molecular properties of the GAPDH preparations in terms of homogeneity (determined by SDS-PAGE) and native quaternary structure (determined by high-performance gel filtration) are summarized in Figure 3 and Figure 4, respectively. Similar to ScGAPDH [48], native NgGAPDH appeared to be a tetrameter of ca. 36 kDa subunits. The recombinant CaGAPDH expressed the same subunit molecular mass, but under non-denaturing conditions, beside the predominant tetrameric form, marked proportions of dimers and monomers were also present. 

### 2.4. Interactions of VTR and HPG with Purified GAPDH

**Figure 5:**
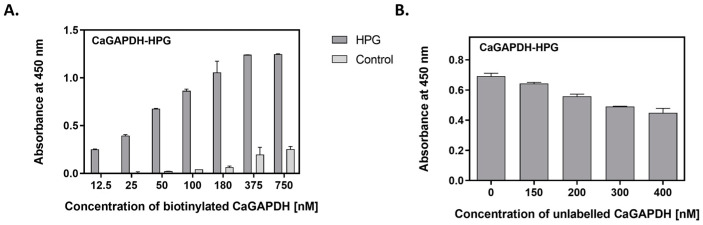
Interactions between CaGAPDH and HPG as measured by microplate ligand-binding assays. HPG was immobilized in microplate wells (3 pmoles per well) by overnight incubation at 4 °C, followed by blocking with bovine serum albumin (BSA). (**A**) Saturable binding assay. Aliquots of biotin-labeled GAPDH at increasing concentrations (50 μL/well) were added to the HPG-coated wells, followed by incubation for 1.5 h at 37 °C. After washing, the adsorbed biotin-labeled protein was detected with the SA-HRP/TMB system. Wells without HPG (only blocked with BSA) served as controls. (**B**) Competitive binding assay. Aliquots (50 μL) of biotin-labeled CaGAPDH at a concentration of 200 nM and unlabeled CaGAPDH at an increasing concentration of 0–400 nM were added to the HPG-coated wells, followed by incubation for 1.5 h at 37 °C. After washing, the adsorbed biotin-labeled protein was detected with the SA-HRP/TMB system.

To further study the interactions of GAPDH with human proteins (VTR, HPG), direct binding studies were conducted using the purified GAPDH proteins. Three different methods were utilized. In the first—semiquantitative microplate ligand-binding assay—VTR or HPG were immobilized in the microplate well and tested for binding to biotin-labeled GAPDH preparations. For both GAPDH preparations, binding to the ECM proteins was dose-dependent. A representative binding plot for CaGAPDH-HPG is presented in Figure 5A, with the other three pairs shown in Figure S1 of Supplementary Materials. Another competitive version of this ligand-binding assay was performed to estimate the actual contribution of direct protein–protein interactions to the total GAPDH binding to VTR- or HPG-coated microplate wells. Here, binding of a fixed concentration of biotin-labeled GAPDH was measured in the presence of an increasing concentration of unlabeled protein (Figure 5B and Figure S1B in Supplementary Materials).

**Figure 6:**
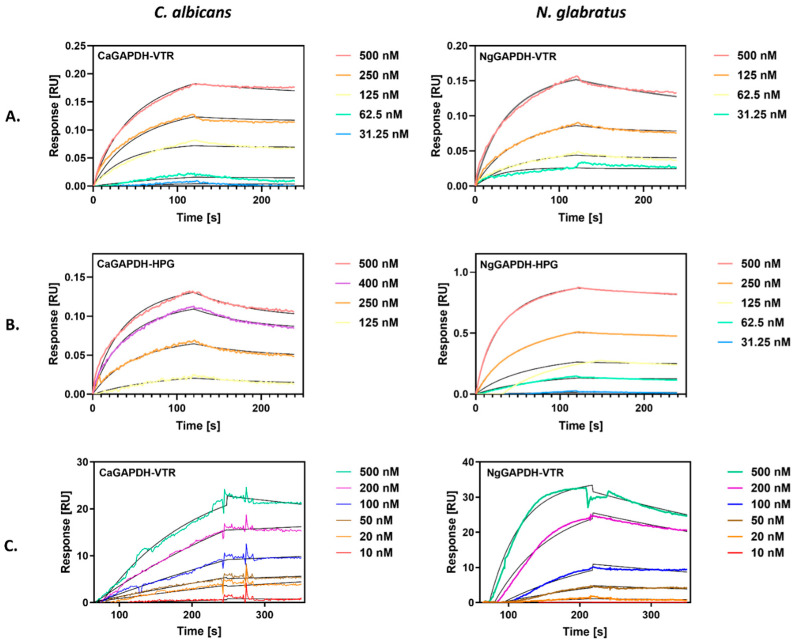
Quantitative analysis of the interactions between candidal GAPDH and human proteins (VTR and HPG) by bio-layer interferometry (BLI) (**A**,**B**) or surface plasmon resonance (SPR) (**C**). (**A**,**B**) GAPDH was immobilized on a BLI AR2G biosensor and analyzed for interactions with solutions of VTR (**A**) or HPG (**B**) at concentrations ranging from 31 nM to 500 nM, using an Octet N1 1.4 system. A Langmuir 1:1 binding model was fitted to the sensograms obtained. (**C**) GAPDH was immobilized on the CM5 SPR sensor chip of a Biacore 3000 system. Solutions of VTR at concentrations in the range of 10–500 nM were injected over the chip at a flow rate of 30 µL/min for 4 min (CaGAPDH) and 3 min (NgGAPDH) for the association and 3 min for the dissociation. A Langmuir 1:1 binding model with baseline drift was fitted to the sensograms obtained.

A second quantitative characterization of the interactions between VTR/HPG and GAPDH from *C. albicans* or *N. glabratus* was performed using bio-layer interferometry (BLI), which allowed the determination of kinetic binding parameters—the association (*k_a_*) and dissociation (*k_d_*) rate constants and the equilibrium dissociation constant (*K_D_ = k_d_*/*k_a_*). In this method, GAPDH was immobilized on the tip of an AR2G sensor, which was then immersed in solutions of the two human proteins. Representative BLI sensograms for the interactions of two GAPDH preparations with VTR and HPG are shown in Figure 6A and Figure 6B, respectively. 

We also attempted to use a more common “reference” method for the quantitative characterization of protein–protein interactions—surface plasmon resonance (SPR) measurements. In this method, GAPDH was immobilized on a CM5 chip, over which solutions of human protein were flowed, in contrast to the static conditions of the BLI method. SPR sensograms were obtained for VTR binding to CaGAPDH and NgGAPDH, as shown in Figure 6C. 

The binding parameters obtained by fitting a Langmuir 1:1 binding model to best quality BLI and SPR sensograms are presented in Table 2. The equilibrium dissociation constants for the interactions of GAPDH with VTR were in an order of 10^−8^ M, as consistently determined by both methods, and of 9 × 10^−9^ M and 1.5 × 10^−7^ M by BLI for HPG binding to NgGAPDH and CaGAPDH, respectively. 

The “moonlighting” interactions of GAPDH with human proteins did not affect the basic, i.e., enzymatic GAPDH activity (Figure S2 in Supplementary Materials), suggesting that the binding site for the non-canonical proteinaceous ligands was well spaced from the catalytic site on the GAPDH molecule. 

Taken together, our study strongly supports a hypothesis that GAPDH, moonlighting on the candidal cell surface, can tightly interact with different host proteins, thus contributing to pathogen adhesion (via VTR binding) or exerting a deregulatory effect (via HPG binding), with both phenomena important for effective infection. 

### 2.5. Adsorption of Exogenous GAPDH to the Surface of Intact C. albicans or N. glabratus Cells

**Figure 7:**
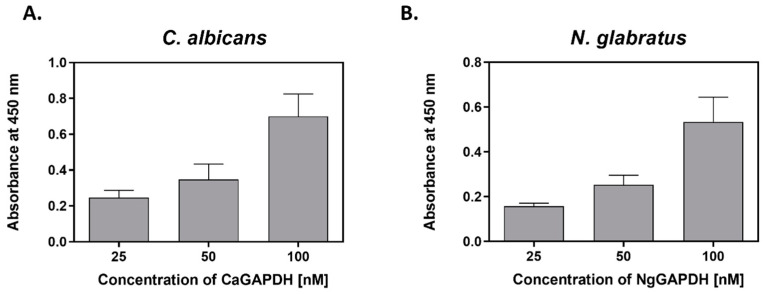
Binding of biotin-labeled GAPDH to *C. albicans* filamentous forms or *N. glabratus* yeast-like cells. (**A**) *C. albicans* hyphae (1 × 10^6^ cells per well) or (**B**) *N. glabratus* blastospores (3 × 10^7^ cells per Eppendorf tube) were cultured in RPMI 1640 medium for 3 h at 37 °C. After external addition of CaGAPDH or NgGAPDH, respectively, followed by incubation and washing, the cell-adsorbed biotinylated protein was detected with the SA-HRP/TMB system.

At infection foci, many microbial proteins, originally intracellular, are likely to be present in a soluble form in the fluid extracellular phase, e.g., due to pathogen lysis [16,49,50] or release from extracellular vesicles [28,51,52,53]. Adsorption of these proteins to the pathogen cell surface may be one explanation for the presence of moonlighting proteins on bacterial or fungal cells [54,55,56,57]. To address this hypothesis for candidal GAPDH, we analyzed the adherence of purified GAPDH to live *C. albicans* and *N. glabratus* cells cultured in RPMI 1640 medium, i.e., existing in filamentous (hyphae) or yeast-like forms, respectively. First, a concentration-dependent cell binding of biotin-labeled CaGAPDH and NgGAPDH was observed (Figure 7).

**Figure 8:**
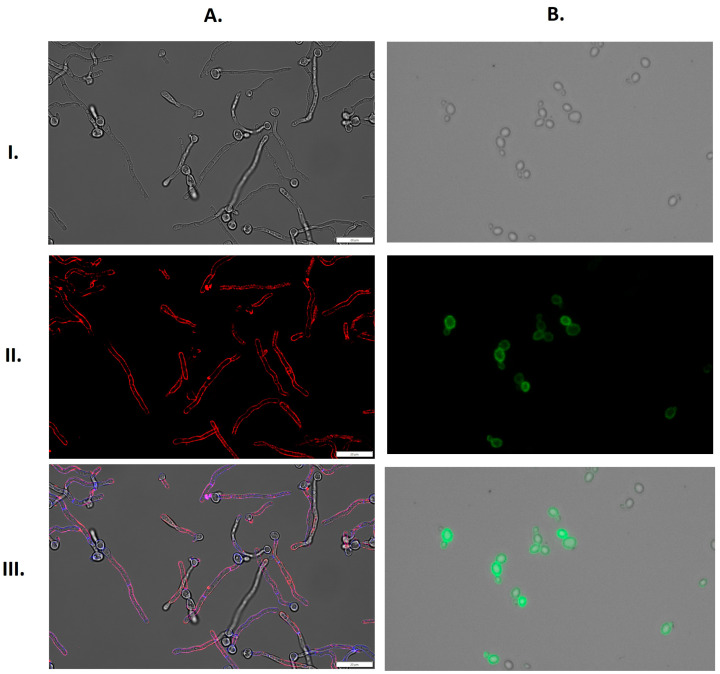
Binding of fluorescently-labeled GAPDH to the surface of filamentous forms of *C. albicans* or yeast-like cells of *N. glabratus*. (**A**) *C. albicans* (1 × 10^6^ cells per well) or (**B**) *N. glabratus* (3 × 10^7^ cells per Eppendorf tube) were cultured in RPMI 1640 medium for 3 h at 37 °C. CaGAPDH was labeled with Alexa 555 (red), and the cell wall of filamentous *C. albicans* was stained with Calcofluor White (blue). NgGAPDH was labeled with fluorescein (green). Images were taken with an Olympus IX73 microscope in bright field (**I**), in the channels TRITC (**A**(**II**)) and FITC (**B**(**II**)) and as an overlay of bright field, TRITC and DAPI (**A**(**III**)) or bright field and FITC (**B**(**III**)). Scale bars: 20 µm.

In addition, GAPDH adsorption was visualized by fluorescence microscopy. Images obtained on an Olympus IX73 microscope showed a strong signal from fluorescently-labeled GAPDH that bound to the filamentous forms of *C. albicans* and yeast-like cells of *N. glabratus*, both cultured in RPMI 1640 medium (Figure 8).

### 2.6. Interactions of GAPDH with the Main Adhesins of C. albicans (Als3) or N. glabratus (Epa6)

**Figure 9:**
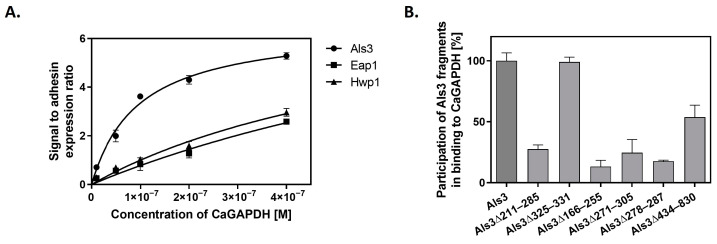
Interaction of CaGAPDH with major *C. albicans* adhesin Als3. (**A**) *S. cerevisiae* mutants with overexpressed and surface-presented Als3, Hwp1 or Eap1 adhesins were mixed with solutions of biotin-labeled CaGAPDH at increasing concentrations in 1.5 mL Eppendorf tubes (3 × 10^7^ cells per tube), and the amount of cell-adsorbed biotinylated GAPDH was estimated using the SA-HRP/TMB system. A one-site specific binding model was fitted to the experimental points using GraphPad Prism 5.01 software. (**B**) For mapping regions of the Als3 molecule involved in CaGAPDH binding, *S. cerevisiae* cells expressing full-length Als3 adhesin or its specified deletion mutants were incubated in 1.5 mL Eppendorf tubes (3 × 10^7^ cells per tube) with biotin-labeled CaGAPDH (100 nM), followed by detection of cell-bound biotinylated CaGAPDH with the SA-HRP/TMB system. Binding levels shown are relative to those for the interaction of CaGAPDH with full-length Als3, which was set at 100%.

In an attempt to identify candidal cell surface proteins that could be “docking platforms” for GAPDH adsorption, we applied an approach similar to that used in our earlier work on another candidal moonlighting protein—*C. albicans* enolase [11]. We used *S. cerevisiae* mutant strains expressing three major *C. albicans* adhesins—Als3, Eap1 (epithelial adhesin protein 1) and Hwp1 (hyphal wall protein 1) [58] (Table S1 in Supplementary Materials). These adhesins are key components of the *C. albicans* cell wall that mediate biofilm formation and adhesion to host epithelial and endothelial cells and other tissues during infection [59,60]. Their ability to interact with fungal GAPDH was analyzed by SA-HRP/TMB detection of biotin-labeled CaGAPDH bound to the cells. As presented in Figure 9A, the GAPDH-binding capacity of Als3-carrying yeast cells was at least two-fold higher than that of cells with overexpressed Eap1 or Hwp1. Moreover, fitting the saturation binding plots to a simple one-site binding model allowed estimated dissociation constants to be calculated as 9.59 × 10^−8^ M, 1.25 × 10^−6^ M and 7.15 × 10^−7^ M for the complexes of CaGAPDH with Als3, Eap1 and Hwp1, respectively. These results strongly suggest that Als3 predominates over the other major *C. albicans* adhesins in their hypothetical roles as binding platforms for GAPDH.

Additionally, we performed experiments aiming to map the regions of the Als3 molecule that contribute to CaGAPDH binding. For this purpose, we used strains of *S. cerevisiae* that overexpress Als3 with specific regions of the protein deleted. Biotin-labeled CaGAPDH was added to these Als3 deletion strains at a constant concentration. Binding to full-length Als3 protein was used as a “total binding” reference (100%), with binding levels to the mutated Als3 proteins presented relative to this (Figure 9B). It was found that only deletion of the amyloid region of Als3 (Δ325–331) did not affect binding to CaGAPDH, while for deletions of the Ig-like N-terminal region (Δ211–285; Δ166–225; Δ271–305; Δ278–287) and the central repeat domain (Δ434–830), attachment to CaGAPDH was significantly decreased. These findings suggest that CaGAPDH binds to multiple sites on the Als3 molecule, with the exception of the amyloid region. For other Als3 fragments, the effects of the deletion may result from their actual interactions with GAPDH, although a possibility of significant conformational damages of Als3 molecule caused by the deletion cannot be excluded.

**Figure 10:**
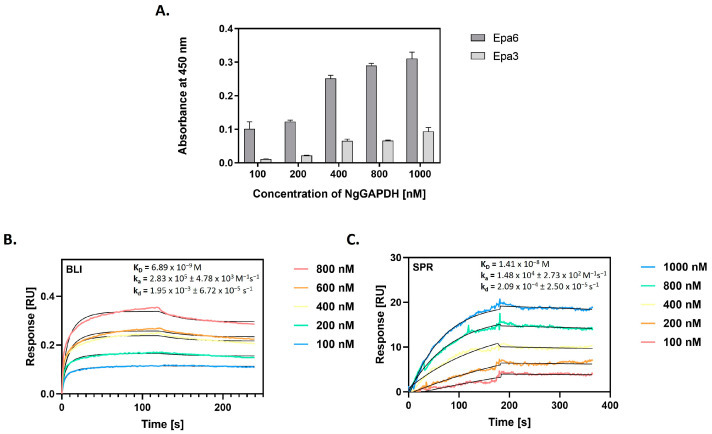
Interaction of NgGAPDH with major *N. glabratus* adhesins Epa6 and Epa3. (**A**) NgGAPDH binding to two major *N. glabratus* adhesins from the Epa family was analyzed using a microplate ligand-binding assay with the SA-HRP/TMB detection system. The microplate wells were coated with isolated and purified Epa6 or Epa3 proteins (5 pmoles per well) by an overnight incubation at 4 °C. Solutions (50 μL) of biotin-labeled NgGAPDH at concentrations within a range of 0–1000 nM were added to the wells, followed by incubation for 1.5 h at 37 °C. As a control, a well blocked with 3% BSA without fungal adhesin was used. (**B**) Sensograms for NgGAPDH binding to Epa6, immobilized on the AR2G biosensor of an Octet N1 1.4 bio-layer interferometry (BLI) system. (**C**) Sensograms for NgGAPDH binding to Epa6, immobilized on a CM5 chip of the surface plasmon resonance (SPR) Biacore 3000 system. In (**B**,**C**), the interactions were measured over NgGAPDH concentrations ranging from 100 to 1000 nM. A Langmuir 1:1 binding model (with baseline drift in (**C**)) was fitted to the sensograms and the kinetic and equilibrium binding parameters were determined, as specified in the plots.

In *N. glabratus*, the main adhesins belong to the Epa (epithelial adhesin) family of cell surface proteins, encoded by 20–25 *EPA* genes, of which two—Epa6 and Epa3 proteins—play predominant roles in mediating adhesion to host epithelial cells [61,62]. The capacity for Epa6 and Epa3 to interact with NgGAPDH was first analyzed using the microplate ligand-binding assay, in which biotin-labeled NgGAPDH was tested for adsorption to microplate wells coated with isolated and purified Epa6 or Epa3 preparations. Saturable NgGAPDH binding was confirmed for both adhesins, but the capacity was at least five-fold higher for Epa6 (Figure 10A). Further quantitative characterization of NgGAPDH-Epa6 interaction was performed with BLI (Figure 10B) and SPR (Figure 10C), which allowed determination of the kinetic and equilibrium binding constants. A dissociation constant in the order of 10^−8^ M was calculated, suggesting a tight interaction between soluble NgGAPDH and surface-adhered Epa6 adhesin.

### 2.7. Effects of CaGAPDH on C. albicans Adhesin Als3 Interactions with Human Proteins VTR and HPG 

**Figure 11:**
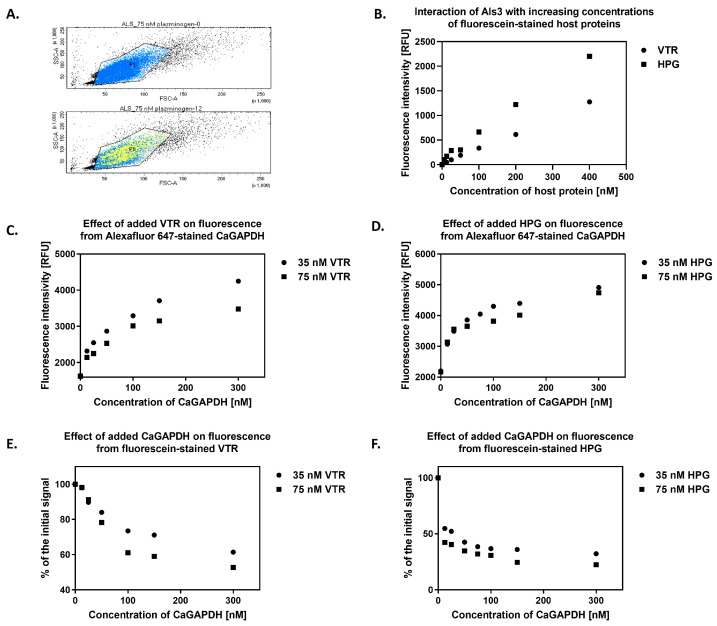
Mutual interactions between CaGAPDH, Als3 and host proteins VTR or HPG. Cells of *S. cerevisiae* UB2416 expressing surface-exposed Als3 lacking the amyloid-forming region (3 × 10^6^ cells per 1.5-mL Eppendorf tube) were incubated with fluorescein-stained human proteins—VTR and HPG—at two concentrations (35 nM or 75 nM) and AlexaFluor 647-stained CaGAPDH at a concentration range of 0–300 nM for 1 h at 37 °C with shaking at 170 rpm. After gentle washing of the cells, flow cytometry analysis was performed using fluorometric detection. The signal was corrected for the baseline, i.e., the signal from cells not expressing Als3 on their surface (strain UB2155). (**A**) The population (FSC, SSC) of the analyzed *S. cerevisiae* cells (P1). Yellow represents the signal from the AlexaFluor 647-stained CaGAPDH. The top diagram shows cells in the absence of CaGAPDH and the bottom diagram with CaGAPDH at a concentration of 12 nM. (**B**) Binding of VTR and HPG to Als3 was performed under the same experimental conditions by adding fluorescein-labeled host proteins at a concentration range of 0–400 nM to *S. cerevisiae* cells, incubating for 1 h at 37 °C with shaking at 170 rpm. After that, flow cytometric analysis was performed, as described above. The diagram shows the correlation of signal intensity from CaGAPDH with its increasing concentration in the presence of host proteins (**C**) VTR or (**D**) HPG. Loss of signal intensity from host proteins (**E**) VTR or (**F**) HPG with an increasing concentration of exogenous CaGAPDH is presented. During flow cytometry analyses, the signal was collected by counting up to 30,000 cells from each sample, the mean of which represents points in the graphs.

As the major candidal adhesins are known to interact with host ECM and blood proteins such as VTR and HPG [27,57,59,62,63,64,65,66], an important issue to address, at least on a preliminary level, was how GAPDH affects the adhesive functions of these cell surface adhesins. For this, we applied flow cytometry (FC) and used an *S. cerevisiae* strain expressing Als3 with the amyloid region deleted (strain UB2416, see Table S1 in Supplementary Materials), which retained the same CaGAPDH-binding properties as the strain carrying full-length Als3 (see above). Removal of the amyloid region eliminated potential problems with cell aggregation, which would otherwise preclude the use of FC. First, the cells were incubated with fluorescein-labeled human proteins at two concentrations (35 nM or 75 nM) and Alexa Fluor 647-labeled CaGAPDH at a concentration range of 0–300 nM. It was observed that the signal from the fluorescently-labeled GAPDH correlated with the concentration (Figure 11A,B) and, in each case, it was lower in the presence of a higher concentration of host protein, suggesting competition between CaGAPDH and human protein for binding to Als3. Thus, it can be concluded that with an increasing concentration of added host proteins, the binding of CaGAPDH to Als3 decreases (Figure 11C,D). In addition, it was observed that the signal from the labeled host proteins decreased with an increasing concentration of exogenous CaGAPDH, implying displacement of the host protein from the complex with Als3 (53–61% for VTR and 22–32% for HPG). It can be inferred that, as the concentrations of added CaGAPDH increase, there is a corresponding decrease in the binding of host proteins to Als3 (Figure 11E,F). 

## 3. Discussion

Over the last decade of research, a large body of evidence has been collected to indicate that numerous pathogenic microorganisms, including bacteria, protozoa and fungi, carry on their cell surfaces multiple moonlighting proteins that play various roles in supporting pathogenesis [5,6,67]. The ability of these multifunctional proteins to interact with proteinaceous components of the ECM [18,25,68], blood coagulation factors [27,69,70] and proteins of the complement system [26] serves to promote pathogen adhesion to host cells and thus contributes to the initiation of infection. The multifunctionality of GAPDH has been confirmed for different organisms [5,71], but a moonlighting role for this glycolysis enzyme in pathogenic yeast-like fungi that until recently had been classified within the single *Candida* genus has not been explored. Nonetheless, the presence of GAPDH on the surface of *C. albicans*, as well as some *Candida* non-albicans species, including *N. glabratus* (formerly *C. glabrata*), has been suggested [5,25], and in vitro interactions of GAPDH with several human proteins such as fibronectin, laminin, plasminogen and factor H have been demonstrated [25,26,27]. 

Our current study focused on the potential for moonlighting GAPDH on two candidal species: *C. albicans*, the most common causative agent of human candidiasis, and *N. glabratus*, which uses slightly different virulence strategies [31] and in some geographic regions compares to *C. albicans* in terms of relative contribution to severe fungal infections [37,38]. Our initial experiments aimed to directly detect GAPDH on the surface of candidal cells following culture in RPMI 1640 medium at 37 °C. This well-buffered defined medium is rich in salts, glucose, vitamins and amino acids, and is appropriate for culturing many types of mammalian cells [72]. Therefore, such growth conditions reflect, in part, the environment found at certain sites of candidal infection [73]. Under these conditions, *C. albicans* forms hyphae, considered the most invasive form of this fungus [73]. With fluorescently-stained anti-ScGAPDH antibodies (confirmed to cross-react with candidal GAPDH), GAPDH was directly visualized on *C. albicans* cells and, moreover, seemed to be located on the filament walls (Figure 1A), thus suggesting specific adsorption and the potential to contribute to candidal pathogenesis. *N. glabratus* can grow only in yeast-like form but is still expected to adjust its cell surface protein profile according to environmental conditions [30]. GAPDH was also visualized on the cells of this candidal species using the anti-ScGAPDH antibodies. Additionally, using Western immunoblotting with the same antibodies, GAPDH was frequently found to be associated with the cell wall of both species (Figure 1B).

As potential non-canonical host proteinaceous ligands for candidal GAPDH, we tested two human proteins whose binding to the fungal cells could significantly influence candidal infections. The adsorption of VTR [63] and HPG [27] to candidal cells has been known for some time, but, in the current work, we demonstrate for the first time the contribution made by GAPDH to this process, albeit at a relatively low level (12–16% for VTR and 19–36% for HPG) (Figure 2). Predictably, other than GAPDH, many other fungal cell surface proteins contribute to the total cellular VTR- and HPG-binding capacity of each species (see below).

For direct confirmation and quantitative analysis of the interactions of candidal GAPDH with VTR and HPG, two preparations of purified GAPDH were obtained. A recombinant CaGAPDH was constructed using the *TDH3* gene sequence from the *C. albicans* genome, and expression was carried out in the *E. coli* strain *Rosetta2* system. A pLATE11 plasmid lacking a tag in its sequence was used, as this tag caused GAPDH to precipitate as an insoluble fraction. This resulted in an enzymatically active protein, although heterogenous in terms of quaternary structure. From the *N. glabratus* cells, native GAPDH was isolated either from cell wall preparations or from the cytoplasm. Although both proteins were active and clearly tetrameric enzymes, only the latter had the specific enzymatic activity comparable to that of the commercial ScGAPDH and thus only this one designated as NgGAPDH was used in most of the subsequent experiments. All three candidal GAPDH preparations were of an acceptable electrophoretic purity. To our knowledge, this is the first time that purified GAPDH from any candidal species has been obtained.

The direct interaction between candidal GAPDH and VTR or HPG was first confirmed semi-quantitatively, using a microplate ligand-binding assay in which the given human protein was immobilized onto the plastic surface and a secondary adsorption of biotin-labeled GAPDH was detected. For rigorous quantitative analysis, two sophisticated instrumental methods were applied—BLI and SPR—in which the protein immobilization setup was reversed compared with the above microplate assay, i.e., GAPDH was first covalently linked to AR2G or CM5 sensors, respectively. An important difference between BLI and SPR methods is that the protein–protein binding to be analyzed occurs either under static or flow conditions, respectively. In spite of these technical variations, the kinetic and equilibrium parameters of binding (Table 2) were comparable between methods, host proteins and GAPDH preparations, with the equilibrium binding constants in an order of 10^−8^ M, similar to those reported for the interactions of various human proteins with candidal cell surface proteins—either moonlighting or GPI-anchored adhesins [11,12,13,50,64]. These parameters qualify the GAPDH-VTR and GAPDH-HPG interactions as fairly strong. Interestingly, the enzymatic activity of GAPDH was not affected by VTR or HPG binding, supporting a more general hypothesis that new functions of moonlighting proteins could have been established during the evolutionary process under conditions that allow the main function to remain intact [8].

The interaction with VTR suggests that GAPDH may promote pathogenesis in these species by enhancing adhesion or through other regulatory functions. Albeit occurring also in blood [74], VTR is one of the major ECM components that plays a role in many physiological processes, including tissue remodeling and regeneration, as well as cell adhesion, proliferation and migration [75]. The three-dimensional structure of the matrix itself is a site that not only functions as a scaffold for cells but also provides them with an environment for growth, differentiation, adhesion and communication between other cells [76]. The human ECM is one of the first contacts between a pathogen and a host and, for this reason, its components are often targeted by proteins located on the surface of microorganisms. For VTR, binding by moonlighting proteins has already been reported in the literature, with a good example being *C. albicans* enolase, which was confirmed to interact with VTR with a *K_D_* of 3–5 × 10^−8^ M [50].

HPG circulates in large quantities in blood and is a key component of the fibrinolysis system. It is a precursor of a potent serine protease—plasmin. Due to the involvement of HPG in many cellular processes, the regulation of its activation is strictly controlled. Examples of factors that can activate HPG include tissue plasminogen activator (tPA) and urokinase plasminogen activator (uPA). In turn, negative modulation of plasmin activity occurs with the participation of serine protease inhibitors—serpins—and also through the immobilization of HPG to its receptors and target molecules. Within the native structure of HPG, five kringle domains play an important role in protein–protein interactions [77]. Several moonlighting proteins have been implicated in mediating the adsorption of HPG to the surface of pathogenic cells [26,27,78,79], and this fairly common phenomenon has been shown to contribute to the evasion of the host immune response and promote tissue invasion. One hypothesis explaining this phenomenon is that the pathogen surface proteins bind HPG, followed by its activation to plasmin, which can then degrade the ECM and facilitate pathogen spreading within the host. In addition, HPG binding to microbial cell surface proteins can interfere with the host immune response, e.g., by preventing complement activation. This allows the pathogen to evade removal by the host immune system and establish a persistent infection [80]. It is also possible that candidal GAPDH can affect the fibrinolysis cascade in a more general sense, e.g., by sequestering its soluble components via cell adsorption, resulting in pathway inhibition or, inversely, assembling the entire cascade on the pathogen surface, thereby enhancing fibrinolysis during infection [81]. The potential pathogenic role of HPG has been extensively studied in the context of its interactions with a major candidal moonlighting protein, enolase [50,66,82]. The interaction with HPG was previously reported for *C. albicans* [27] and other microorganisms [80].

Mechanisms of cell surface targeting of microbial moonlighting proteins, including candidal GAPDH, have been unsatisfactorily explored. Multiple protein transport modes have been proposed [50,83], but none have yet to be conclusively proven for any protein. Still, it is also possible that multiple mechanisms are effective for one protein, i.e., that the population of a given cell surface-bound protein comprises molecules that have used different transport pathways. As a rule, the precursors of moonlighting proteins lack any signal peptide that would direct them to the classic secretion path. Thus, several unconventional secretion mechanisms have been postulated to explain the extracellular localization of moonlighting proteins [52,83,84]. Another hypothesis is that moonlighting proteins are adsorbed from the external environment onto growing cells [11,12,16,49]. One explanation for the presence of the soluble form of these multifunctional proteins in the environment outside the cells is by their release from extracellular vesicles [28,52]. The presence of GAPDH in the fraction of *C. albicans* extracellular vesicles [52,85] seems to support this proposal. Another hypothesis is that cytosolic proteins are released during cell lysis. Correlations between the amount of a certain moonlighting protein on the cell surface with the degree of cell lysis have been found in several bacterial species such as for GAPDH in *Streptococcus* spp. [16,49]. Other examples include four cytoplasmic proteins found on the cell wall of *Lactobacillus crispatus*: enolase, GAPDH, glutamine synthetase and glucose-6-phosphate isomerase. Treatment of cells with a peptide that disturbs membrane integrity and a change in pH, resulting in an increase in cell wall permeability, caused an increase in the number of proteins exposed on the pathogen surface [54,86]. The ability of enolase to be adsorbed to the surface of pathogenic cells was shown for *Streptococcus pneumoniae* [55] and *C. albicans* [11]. In *S. cerevisiae* yeast, the species closely related to *N. glabratus*, the secretion of GAPDH protein, which in a hydrolyzed form serves as an antibacterial peptide in the external environment, was demonstrated [24].

The experiments conducted in our current work clearly showed the ability of candidal GAPDH to adsorb to the surface of both *C. albicans* and *N. glabratus* cells (Figure 7). Microscopy images (Figure 8) confirmed the binding of GAPDH to the blastospore cell wall and, again, a strong signal from the walls of *C. albicans* hyphae was observed. These two independent experiments confirmed the capacity for GAPDH to be adsorbed to the candidal cell surface, indicating a possible mechanism by which the extracellular localization of this moonlighting protein could occur.

Another issue addressed in the current work concerned the identification of potential binding sites for GAPDH on *C. albicans* and *N. glabratus* cells. It is possible that GAPDH can interact with different types of cell surface molecule, but, as reported for our recent detailed study on *C. albicans* enolase [11], proteinaceous cell wall components probably predominate in mediating the capacity for candidal cells to bind GAPDH. Multiple enolase-binding proteins were identified in the *C. albicans* cell wall [11], most of which apparently belonged to the moonlighting protein class. It is possible that moonlighting proteins on the pathogen cell surface can form a network of mutually interacting partners. It is known that several enzymes of the glycolysis pathway, including GAPDH, can form complexes with each other in the cytoplasm [87]. However, the major GPI-anchored adhesin—Als3—was also identified as an enolase-binding protein [11]. By analogy, in the current work, we analyzed putative interactions of GAPDH with major *C. albicans* adhesins Als3, Eap1 and Hwp1 and adhesins Epa6 and Epa3 for *N. glabratus*. Using *S. cerevisiae* mutants expressing these three *C. albicans* adhesins on their surface [58], Als3 was found to bind CaGAPDH most strongly, both in terms of binding capacity and the equilibrium binding constant (*K_D_* in an order of 10^−8^ M). As Als3 is known to bind many proteins via multiple sites on its molecule [4], we next attempted to determine which regions were involved in GAPDH binding. Using *S. cerevisiae* strains that expressed Als3 carrying different domain deletions [88], we found that only the amyloid region (Δ325–331) definitely did not contribute to the interaction with CaGAPDH. Thus, the Als3 molecule can provide several different binding sites for GAPDH on the *C. albicans* cell surface.

For the identification of NgGAPDH interactions with major *N. glabratus* adhesins, we applied another approach. Two purified proteins from the Epa family—Epa6 and Epa3—were tested for binding to GAPDH using three methods—microplate ligand-binding assay, BLI and SPR (Figure 10). With the first method, Epa6 was shown to bind strongly to GAPDH. With the two other methods, the Epa6-NgGAPDH interactions were analyzed in terms of kinetic and equilibrium binding parameters, whose values suggested a fairly tight binding between the two proteins (*K_D_* of 7 × 10^−9^ M to 1.4 × 10^−8^ M, respectively).

The data imply that the interactions of GAPDH with Als3 or Epa6 constitute only a part of total GAPDH-binding capacity for *C. albicans* and *N. glabratus*. However, these major adhesins are essential for pathogen adhesion to host cells and tissues and can adhere to numerous host proteins [4,62]. An interesting problem thus arises as to whether moonlighting proteins such as enolase, GAPDH and others enhance the Als3- or Epa6-dependent adhesion of *C. albicans* and *N. glabratus* to host tissues; in other words, whether the moonlighting proteins can form bridges between pathogen and host cells. To address this issue for CaGAPDH, flow cytometry was used to analyze the interactions of mixtures of CaGAPDH-VTR-HPG with *S. cerevisiae* cells expressing Als3 on their surface (Figure 11). Unfortunately, during the analysis, only the competition between CaGAPDH and the host proteins was detected. In our previous study, we observed the same effect with another moonlighting protein—enolase [11]. Thus, the “bridging hypothesis” cannot be confirmed at present. The capacity for surface-bound GAPDH to promote or antagonize fungal cell attachment to host proteins mediated by Als3 remains an important area for future study.

## 4. Materials and Methods

### 4.1. Yeast Strains and Culture Conditions

Cells of *N. glabratus* strain CBS138 (ATCC^®^ 2001™) were cultured in a glass flask for 16 h in 20 mL of YPD medium (1% yeast extract, 2% soy peptone and 2% glucose) with shaking at 170 rpm at 30 °C. Then, 1 mL of the cell suspension was added to 20 mL of RPMI 1640-defined medium, pH 7.4, followed by shaking at 170 rpm at 37 °C for 72 h. *C. albicans* (ATCC^®^ 10231™ strain) was cultured in YPD medium for 16 h at 30 °C with shaking at 170 rpm and in RPMI 1640 medium for 72 h at 37 °C with shaking at 170 rpm. *S. cerevisiae* strains listed in Table S1 in Supplementary Materials [58,88] were cultured in a complete synthetic medium (CSM, Formedium, Norfolk, UK) enriched with 0.67% YNB (yeast nitrogen base) and 2% glucose (Sigma-Aldrich, Burlington, MA, USA) for 24 h at 30 °C with shaking at 170 rpm.

### 4.2. Identification of GAPDH on the Surface of C. albicans and N. glabratus Cells

To identify GAPDH on the cell surface of *C. albicans* and *N. glabratus* cells, polyclonal rabbit antibodies against *S. cerevisiae* GAPDH (anti-ScGAPDH), (Hexabiogen, Marrakesh, Morocco) and anti-rabbit antibodies derived from mouse conjugated to alkaline phosphatase or Alexa Fluor 488 (Abcam, Cambridge, UK) were used for Western immunoblotting or fluorescence microscopy, respectively. Mixtures of candidal cell wall proteins (CWP) were isolated using a β-1,6-glucanase enzyme (Takara Bio Inc., Otsu, Shiga, Japan), as described previously [66]. The obtained CWP mixture and a control sample of commercially purchased *S. cerevisiae* GAPDH (Sigma-Aldrich) were subjected to SDS-PAGE under denaturing conditions, followed by electrotransfer onto a methanol-activated polyvinylidene fluoride (PVDF) membrane (Immobilon, Millipore, Burlington, MA, USA) for 90 min at 250 mA in 10 mM CAPS buffer, 10% methanol, pH 11.0 [89]. After transfer, the membrane was blocked overnight at 4 °C with a 5% non-fat milk solution in TTBS buffer (50 mM Tris, 0.5 M NaCl, 0.05% Tween 20, pH 7.4). After blocking, two sequential incubations of the membrane with antibodies—rabbit anti-ScGAPDH and anti-rabbit alkaline phosphatase-conjugated secondary antibodies—were performed, with each incubation conducted for 1 h with gentle shaking. After each step, the membrane was washed three times for 15 min in TTBS buffer. Detection was performed using the substrate for alkaline phosphatase—5-bromo-4-chloro-3-indolyl phosphate/nitroblue tetrazolium (BCIP/NBT) (Sigma-Aldrich).

### 4.3. Binding of VTR and HPG to Candidal Cells in the Presence or Absence of Anti-GAPDH Antibodies 

HPG (Sigma-Aldrich) and VTR (R&D Systems, Minneapolis, MN, USA) were biotin-labeled using biotin N-hydroxysuccinimide ester (NHS-biotin; Sigma-Aldrich) according to the manufacturer’s instructions. A 20-fold molar excess of NHS-biotin in dimethylformamide (DMF) was added per 1 mole of human protein in 0.1 M carbonate buffer at pH 8.3, followed by incubation on ice for 4 h and final dialysis at 4 °C in PBS with two buffer changes. *C. albicans* cells were cultured for 16 h in YPD medium at 30 °C with shaking at 170 rpm, after which the cells were washed with PBS, resuspended in RPMI 1640 medium and seeded (10^6^ cells per well) in the wells of a MaxiSorp 96-well plate (Nunc, Roskilde, Denmark). Cells were incubated at 37 °C for 3 h. After that time, when the cells had developed hyphae, cells were washed three times with 1% BSA in PBS, followed by blocking the well surface with 3% BSA in PBS for 1.5 h at 37 °C. In subsequent steps, 50 µL of anti-ScGAPDH antibodies (1 µg/mL) were added, followed by 50 µL of 150 nM biotin-labeled VTR or biotin-labeled HPG. The incubation lasted for 1 h at 37 °C, and, after each step, cells were washed three times with 1% BSA in PBS. The detection was performed using the SA-HRP/TMB system; briefly, cells were incubated for 1 h in the dark with SA-HRP solution and, after rinsing, 50 µL of TMB was added and, after a specified time, the reaction was stopped by adding 50 µL of 2 M HCl. The absorbance was measured at 450 nm using a Power Wave X Select microplate reader (BioTek, Winooski, VT, USA). *N. glabratus* cells were cultured in RPMI 1640 medium for 72 h at 37 °C with shaking at 170 rpm, and the experiment was performed as for *C. albicans*, except that 1.5 mL Eppendorf tubes were used instead of the microplate (3 × 10^7^ cells per tube). After each step, cells were washed three times with 1% BSA in PBS and centrifuged for 3 min at 3000× *g*. Before absorption measurements, cells were transferred to new tubes to minimize non-specific protein–plastic interactions.

### 4.4. Expression and Isolation of Recombinant CaGAPDH

Expression of recombinant CaGAPDH was carried out based on the method described in our previous articles, with some modifications [13,50]. For RNA isolation, *C. albicans* cells were cultured in YPD medium to an early logarithmic phase (OD600 = 0.4), after which 6 mL of culture was washed three times with PBS and centrifuged for 3 min at 3000× *g*. The pellet was frozen in liquid nitrogen and, after thawing, resuspended in TRI^®^Reagent (Sigma-Aldrich). Glass beads (425–600 µm, Sigma-Aldrich) were added and the mixture was homogenized using a FastPrep Precellys Evolution device (Bertin Technologies, Montigny-le-Bretonneux, France), performing two homogenization cycles at 6 rpm for 45 s each. Between cycles, the sample was incubated for 2 min on ice. The RNA was extracted according to the protocol provided with a Clean-Up RNA Concentrator (A&A Biotechnology, Gdynia, Poland), and the integrity of the isolated RNA was verified electrophoretically by running an aliquot of the RNA sample on an agarose gel stained with ethidium bromide to see clear 28S and 18S rRNA bands. To synthesize cDNA, 2 µg of RNA was used in the PCR reaction, which was carried out using 0.5 g of oligonucleotide primer (dT)18 and 200 U of MLV reverse transcriptase (Moloney murine leukemia virus reverse transcriptase; Promega, Madison, WI, USA). The GAPDH coding region of *C. albicans* was amplified using a C1000 Touch Thermal Cycler (Bio-Rad, Hercules, CA, USA) and using primers designed according to the instructions provided with the aLICator LIC Cloning and Expression Kit (Thermo Fisher Scientific, Waltham, MA, USA) for plasmid pLATE11: forward primer 5′AGAAGGAGATATAACTATGATGGCTATTAAAATTGGTATT3′ and reverse primer: 5′GGAGATGGGAAGTCATTATCAAGCAGAAGCTTTAGC3′. The reaction conditions were as follows: 95 °C for 2 min, followed by 30 cycles of 95 °C for 30 s, 56 °C for 30 s, 72 °C for 3 min and 72 °C for 10 min. The PCR products were electrophoretically verified on a 1% agarose gel and purified using the Gel-Out Concentrator Kit (A&A Biotechnology), according to the manufacturer’s instructions, before being incorporated into the pLATE11 plasmid. Using the aLICator LIC Cloning and Expression Kit, the insert released cDNA was processed with the aLICator LIC Cloning and Expression Kit (Thermo Scientific Fischer). After 5 min of incubation of the PCR product with T4 DNA polymerase in LIC buffer, the reaction was stopped by adding EDTA to a final concentration of 30 nM, followed by addition of the pLATE11 expression vector and incubation for 5 min. The insert construct annealed mixture was used directly to transform *E. coli* TOP10 competent cells (Thermo Fisher Scientific), which were incubated on ice for 30 min, after which time the mixture was exposed to 42 °C for 45 s and then put back on ice for 5 min. Lysogeny broth (LB) was added to the reaction mixture and shaken at 180 rpm for 1 h at 37 °C, after which cells were seeded onto an LB plate with ampicillin (100 µg/mL). Positive colonies were selected for their resistance to ampicillin and then the plasmids were isolated using the Mini Plasmid Kit (A&A Biotechnology). The correct sequences were verified by sequencing (Genomed, Warsaw, Poland), and then plasmid DNA (200 ng) was used to transform chemically competent Rosetta^TM^ 2 (DE3) *E. coli* cells. Transformants were recovered on tryptic soy broth (TSB) plates supplemented with ampicillin (100 µg/mL) and chloramphenicol (34 µg/mL). Positive clones were cultured in TSB and incubated at 37 °C with shaking at 180 rpm until the early logarithmic phase. After that time, the culture was induced with isopropyl-β-D-thiogalactoside (IPTG) at a final concentration of 1 mM, the temperature of incubation was reduced to 20 °C and the culture was shaken overnight. The cells were then resuspended in lysis buffer (50 mM NaH_2_PO_4_, 300 mM NaCl, 10 mM imidazole, 20% glycerol), sonicated in a Sonic Ruptor 400 Ultrasonic Homogenizer (Omni International, Kennesaw, GA, USA) (6 min, amplitude 0.5) and centrifuged at 20,000× *g* for 30 min at 4 °C. The supernatant was subjected to a two-step purification as described by Schmalhausen et al. (2019) [90]. In the first step, the protein mixture was fractionated by precipitation with ammonium sulfate. For this, ammonium sulfate was added to the supernatant in small portions at 4 °C with constant stirring until 50% saturation was reached. After 2 h, the precipitate was separated by centrifugation at 15,000× *g* for 15 min, and the supernatant was likewise brought to 70% saturation and centrifugation was repeated after 30 min of stirring. The supernatant was this time brought to 85% saturation and left at 4 °C with constant stirring overnight. The next day, the suspension was centrifuged at the same parameters and the resulting precipitate was resuspended in a buffer (50 mM HEPES, 2 mM DTT, pH 7.0) and dialyzed against it for 48 h with two buffer changes. The second purification step involved ion exchange chromatography. The solution was injected onto a ResourceQ 1 mL column (GE Healthcare, Uppsala, Sweden) and separation was carried out using a linear gradient of 0–0.2 M NaCl (20 mL) at a flow rate of 1 mL/min. After each purification step, the purity of the selected fractions and the presence of GAPDH were verified by SDS-PAGE, and the enzymatic activity of GAPDH was analyzed using a commercial activity assay kit (Sigma-Aldrich).

### 4.5. Isolation and Purification of N. glabratus GAPDH

GAPDH present on the surface of *N. glabratus* cells (S-NgGAPDH) was isolated using the cell wall protein isolation protocol described previously [66]. The mixture of CWP was dialyzed against 20 mM Tris, pH 8.0, at 4 °C, with two buffer changes. The solution was applied to a ResourceQ 1 mL column pre-equilibrated with the same buffer and the separation was carried out in a linear gradient of 0–0.5 M NaCl (20 mL) at a flow rate of 1 mL/min. The fraction was further purified on a TSK G 3000 SW column (21.5 mm × 30 cm, particle size 13 µm) (Tosoh Bioscience, King of Prussia, PA, USA), which was pre-equilibrated with 0.1 M sodium sulphate, 0.1 M sodium dihydrogen phosphate, pH 6.7. 

Cytoplasmic *N. glabratus* GAPDH (NgGAPDH) was isolated from *N. glabratus* cells cultured in YPD medium for 16 h at 30 °C with shaking at 170 rpm. Cells were washed three times with PBS. Then, protease inhibitor mixture (Complete Tablets EDTA-free, EASYpack, Roche, Penzberg, Germany) was added, followed by sonication (3 cycles of 3 min each, amplitude 0.8) using a Sonic Ruptor 400 ultrasonic homogenizer (Omni International). The disrupted cells were centrifuged at 15,000× *g* for 30 min, and ammonium sulfate was added in small portions to the supernatant at 4 °C with constant stirring until 40% saturation was reached. After one hour, the suspension was centrifuged at 15,000× *g* for 15 min, and the supernatant was brought to 90% ammonium sulfate saturation. The suspension was again centrifuged, and the precipitate was dissolved in 50 mM Tris, 1 mM EDTA, 0.2 mM DTT, pH 7.5, and dialyzed against it at 4 °C with two buffer changes. The resulting protein mixture was separated on a MonoQ 1 mL column (Pharmacia Biotech, Uppsala, Sweden) pre-equilibrated with the same buffer. After sample injection, the separation was carried out in a linear gradient of 0–0.5 M NaCl (60 mL) at a flow rate of 1 mL/min. The fraction containing GAPDH was subjected to a further purification step on a TSK G 3000 SW column (21.5 mm × 30 cm, particle size 13 µm), which was pre-equilibrated with 0.1 M sodium sulfate, 0.1 M sodium dihydrogen phosphate, pH 6.7. After each step of GAPDH purification, the obtained fractions were analyzed by SDS-PAGE, and enzyme activity was measured using the GAPDH activity assay kit (Sigma-Aldrich).

### 4.6. GAPDH Activity Assay

The GAPDH activity assay was carried out according to the manufacturer’s protocol (GAPDH Activity Assay Kit, Sigma-Aldrich, cat. no. MAK277). Briefly, a sample containing GAPDH was added to the well of a MaxiSorp 96-well plate (Nunc) in GAPDH Assay Buffer, to which Master Reaction Mix containing GAPDH Developer and GAPDH Substrate in GAPDH Assay Buffer was added. The reaction was carried out for 30 min at 37 °C, measuring absorbance at 450 nm every 1 min. GAPDH activity was recalculated based on the NADH standard curve, where one unit (U) corresponded to the amount of GAPDH enzyme that generated 1 µmol of NADH per minute at pH 7.2 at 37 °C.

### 4.7. Gel Filtration Analysis of GAPDH Quaternary Structure

For estimation of native GAPDH molecular mass, and thus its oligomerization state, different forms of GAPDH were analyzed by gel filtration on a Superdex 200 HR 10/50 column (Amersham Biosciences, Uppsala, Sweden) pre-calibrated with a protein molecular mass standard mixture (Bio-Rad), with samples eluted using 20 mM Tris-HCl pH 6.7 buffer at a flow rate of 0.5 mL/min. GAPDH samples were centrifuged at 15,000× *g* for 15 min before injection onto the column, and the chromatography was performed with absorbance monitoring at 280 nm. 

### 4.8. Microplate Ligand-Binding Assay of the Interactions of GAPDH with Human VTR and HPG

Biotinylation of CaGAPDH and NgGAPDH was performed using the same method as described above for VTR and HPG. VTR and HPG solutions in PBS (50 µL, 3 pmol per well) were added to the wells of MaxiSorp 96-well plates (Nunc) for overnight incubation at 4 °C. The plate wells were then washed three times with 1% BSA in PBS, blocked with 3% BSA in PBS for 1.5 h at 37 °C and washed again. Then, solutions of biotin-labeled GAPDH proteins at concentrations in a range of 0–600 nM were added, followed by incubation for 1 h at 37 °C. After the unbound proteins were washed off, detection of adsorbed GAPDH was performed using the SA-HRP/TMB system, with absorbance measurements at 450 nm using the Power Wave X Select microplate reader. In another competitive assay version, mixtures of biotin-labeled GAPDH at a fixed concentration of 200 nM for CaGAPDH or 300 nM for NgGAPDH were mixed with unlabeled GAPDH at 0–600 nM and added to microplate-immobilized human proteins. Further assay steps were performed as described above.

### 4.9. Bio-Layer Interferometry (BLI) Analysis of the Interactions of GAPDH with VTR and HPG

BLI measurements were performed with an Octet N1 1.4 apparatus (Sartorius, Göttingen, Germany). GAPDH was immobilized in 10 mM HEPES, pH 7.4 on an AR2G biosensor (Sartorius) that had been activated with a mixture of 20 mM 1-ethyl-3-(3-dimethylaminopropyl)carbodiimide (EDC) and 10 mM N-hydroxysuccinimide (NHS) (Sartorius). GAPDH solution was prepared at a concentration of 30 µg/mL in 10 mM acetate buffer, pH 4.5. After exposing the protein to the active surface, the sensor was treated with 1 M ethanolamine, pH 8.0 (Sartorius). Interactions were measured in accordance with the manufacturer’s recommendations over a concentration range of human proteins between 31 and 500 nM. Sensograms were analyzed using Octet N1 software v. 1.4.0.13, which, on the basis of a Langmuir binding model (1:1), allowed estimation of the kinetic and equilibrium parameters of binding.

### 4.10. Surface Plasmon Resonance (SPR) Analysis of the Interactions of GAPDH with VTR 

SPR analysis was performed using a Biacore 3000 system (GE Healthcare). CaGAPDH and NgGAPDH in 10 mM HEPES, pH 7.4 were immobilized via amine residues using the Amine Coupling Kit (GE Healthcare) on the surface of a CM5 chip (GE Healthcare) that had been activated with 50 mM EDC and 200 mM NHS. GAPDH solutions were injected at a concentration of 10 µg/mL in 10 mM sodium acetate, pH 4.5 at a flow rate of 10 µL/min. For analysis, an HBS buffer (10 mM HEPES, 150 mM NaCl, 0.005% P20 (GE Healthcare), pH 7.4) was used. The interactions of GAPDH with human VTR or HPG at concentrations of 10–500 nM were studied at a flow rate of 30 µL/min at 25 °C. The chip surface was regenerated after each measurement with 1 M NaCl. Sensograms were analyzed using BiaEvaluation v. 4.1.1 software, which, on the basis of a Langmuir 1:1 model (with baseline drift), allowed determination of the kinetic and equilibrium parameters of binding.

### 4.11. Analysis of GAPDH Adsorption on Candidal Cells 

GAPDH adsorption to the filamentous form of *C. albicans* was studied using a MaxiSorp 96-well plate (Nunc), into which 1 × 10^6^ cells per well were seeded in RPMI 1640 medium, followed by incubation of the plate for 3 h at 37 °C. Wells were then blocked with 3% BSA in PBS buffer for 1.5 h, and biotin-labeled CaGAPDH was added to the wells for a further 1 h. Detection of adsorbed biotinylated CaGAPDH was performed with SA-HRP/TMB, with absorbance measurements at 450 nm. Wells were washed three times with 1% BSA in PBS after each step. For the yeast-like form of *N. glabratus*, the experiment was conducted similarly, but in 1.5 mL Eppendorf tubes containing 3 × 10^7^ cells. All incubations were performed with shaking at 170 rpm. Prior to detection, cell suspensions were transferred to new tubes. 

To visualize GAPDH adsorption on candidal cells, CaGAPDH was labeled with Alexa 555 fluorescent dye (Thermo Fisher Scientific) and NgGAPDH with NHS-fluorescein (Sigma-Aldrich), following the manufacturers’ protocols. Briefly, the Alexa 555 dye was added to CaGAPDH in a 20-fold molar excess and the mixture was incubated for 1 h at 37 °C with shaking at 170 rpm. A solution of NHS-fluorescein in DMF (20-fold molar excess of reagent) was added to NgGAPDH at the same proportions, followed by incubation in the dark at 4 °C for 2 h. After labeling, the solutions of both proteins were dialyzed against PBS at 4 °C for 48 h with two buffer changes. *C. albicans* cells were seeded onto a glass-like microplate (CellVis, Mountain View, CA, USA) (1 × 10^4^ cells per well) in RPMI 1640 medium and cultured at 37 °C for 3 h. After blocking with 3% BSA in PBS, cells were incubated for 1 h with Alexa Fluor-555-conjugated CaGAPDH. Additionally, the cell wall of the hyphae was stained for 10 min with 0.1 mg/mL PBS Calcofluor White solution (Fluka, Buchs, Switzerland). After each step, cells were washed three times with 1% BSA solution in PBS buffer. *N. glabratus* cells, cultured in RPMI 1640, were incubated in 1.5 mL Eppendorf tubes (3 × 10^7^ cells per tube) with fluorescein-conjugated NgGAPDH for 1 h with shaking at 170 rpm, and then the cells were washed three times in PBS, centrifuged for 3 min at 3000× *g*, resuspended in PBS and transferred to a glass-like microplate. Both cell types were observed under an Olympus IX73 microscope (Olympus, Tokyo, Japan).

### 4.12. Binding of CaGAPDH to Major C. albicans Adhesins Als3, Eap1 or Hwp1 Expressed on the Surface of S. cerevisiae

*S. cerevisiae* strains UB2155, UB2157, UB2158 and UB2159 (Table S1 in Supplementary Materials) were cultured in complete synthetic medium (CSM) for 24 h at 30 °C with shaking at 170 rpm, washed with PBS by centrifugation for 3 min at 3000× *g* and transferred to 1.5 mL Eppendorf tubes (3 × 10^7^ cells per tube). Biotin-labeled CaGAPDH at concentrations of 0–400 nM was added to the cells, followed by incubation for 1 h at 37 °C with shaking at 170 rpm. After washing the cells three times in PBS, detection of adsorbed CaGAPDH was performed using the SA-HRP/TMB system, with absorbance measurements at 450 nm. Prior to detection, cells were transferred to new tubes to avoid nonspecific protein–plastic interactions. For the Als3 domain deletion strains, an analogous experiment was performed, except that biotinylated CaGAPDH was added at a fixed concentration of 100 nM.

### 4.13. Analysis of NgGAPDH Interactions with N. glabratus Epa6 or Epa3 Adhesins 

The major *N. glabratus* adhesins Epa6 and Epa3 were isolated and purified using the same protocol as for S-NgGAPDH isolation. Their interactions with NgGAPDH were analyzed using three methods. First, was the microplate ligand binding assay, similar to that described in Section 4.8. The microplate wells were coated with Epa proteins (5 pmoles per well) by overnight incubation at 4 °C and then blocked with 3% BSA. Aliquots (50 μL) of biotin-labeled NgGAPDH at a concentration of 0–1000 nM were added to the wells, followed by incubation for 1.5 h at 37 °C, washing and final detection with the SA-HRP/TMB system. The two other quantitative methods were BLI (Section 4.9) and SPR (Section 4.10), for which Epa6 was immobilized on the AR2G biosensor of an Octet N1 1.4 system or CM5 chip of a Biacore 3000 system, respectively. Interactions were measured over a NgGAPDH concentration range of 100–1000 nM. A Langmuir 1:1 binding model (with baseline drift in SPR measurements) was fitted to the sensograms to allow determination of the kinetic equilibrium binding parameters. 

### 4.14. Analysis of the NgGAPDH Interactions with N. glabratus Epa6 and Epa3 Adhesins 

Flow cytometry measurements were performed using *S. cerevisiae* strain UB2316 on a BD LSRFortessa™ cell analyzer (Franklin Lakes, NJ, USA). VTR and HPG proteins were labeled with NHS-fluorescein, as described above for NgGAPDH. The CaGAPDH protein was labeled with Alexa Fluor 647 (Thermo Fisher Scientific) in DMF following the manufacturer’s instructions. A 20-fold molar excess of the labeling reagent was incubated with CaGAPDH for 1 h at 37 °C. The labeled proteins were then dialyzed against PBS at 4 °C for 48 h with two buffer changes. Yeast cells were cultured in CSM medium at 30 °C for 24 h with shaking at 170 rpm, washed with PBS and transferred to 1.5 mL Eppendorf tubes (3 × 10^6^ cells per tube). Fluorescein-labeled VTR or HPG were added to the cells at two concentrations (35 nM and 75 nM), while Alexa Fluor 647-conjugated CaGAPDH was added at a concentration range of 0–300 nM, followed by incubation for 1 h at 37 °C with shaking at 170 rpm. After incubation, cells were washed three times with PBS to remove unbound proteins and resuspended in 0.5 mL PBS, before being transferred to appropriate flow cytometry tubes and analyzed. Fluorescence was measured at excitation and emission wavelengths of 488 nm and 519 nm for fluorescein-labeled VTR and fluorescein-labeled HPG and 633 nm and 660 nm for Alexa Fluor 647-conjugated CaGAPDH, respectively.

## Figures and Tables

**Table 1:** A representative purification table for GAPDH from *N. glabratus* cytoplasm.

Step	Volume (mL)	Total Protein (mg)	Total Activity (mU)	Specific Activity (mU/mg)	Purification (Fold)	Yield (%)
Crude extract	21	23	14,900	552	-	-
Precipitation with ammonium sulfate 40–90%	5	3.94	3410	575	1.04	23.1
Ion-exchange chromatography MonoQ	2	1.27	3250	2520	4.38	22
Gel filtration	8	0.25	2580	5970	10.4	17.4

**Table 2:** Kinetic and equilibrium binding parameters for the interactions of candidal GAPDH with VTR and HPG, as determined by BLI and SPR measurements.

Human Protein	Method	GAPDH	*k_a_* (M^−1^s^−1^)	*k_d_* (s^−1^)	*K_D_* (M)
VTR	BLI	CaGAPDH	(3.89 ± 0.08) × 10^4^	(7.99 ± 0.40) × 10^−4^	(2.06 ± 0.10) × 10^−8^
		NgGAPDH	(5.07 ± 0.06) × 10^4^	(1.72 ± 0.03) × 10^−3^	(3.39 ± 0.06) × 10^−8^
	SPR	CaGAPDH	(1.40 ± 0.02) × 10^4^	(6.19 ± 0.35) × 10^−4^	(4.42 ± 0.25) × 10^−8^
		NgGAPDH	(3.81 ± 0.05) × 10^4^	(1.83 ± 0.02) × 10^−3^	(4.80 ± 0.06) × 10^−8^
HPG	BLI	CaGAPDH	(3.30 ± 0.31) × 10^4^	(4.96 ± 0.16) × 10^−3^	(1.50 ± 0.05) × 10^−7^
		NgGAPDH	(7.46 ± 0.04) × 10^4^	(6.49 ± 0.13) × 10^−4^	(8.69 ± 0.17) × 10^−9^

## Data Availability

The datasets generated and analyzed during the current study are available in the Cracow Open Research Data Repository, https://doi.org/10.57903/UJ/KEK8YB.

## Supplementary Materials

The supporting information can be downloaded at https://www.mdpi.com/article/10.3390/ijms25021013/s1.
