# Supplementary material for: Glyceraldehyde 3-Phosphate Dehydrogenase on the Surface of Candida albicans and Nakaseomyces glabratus Cells—A Moonlighting Protein That Binds Human Vitronectin and Plasminogen and Can Adsorb to Pathogenic Fungal Cells via Major Adhesins Als3 and Epa6
Source: Int J Mol Sci. 2024 Jan 13;25(2):1013. doi: 10.3390/ijms25021013 (PMC10815899; doi:10.3390/ijms25021013)
Supplement: Supplementary file 1 [file ijms-25-01013-s001.zip › Bednarek 2023_supplementary materials - 10-12-2023 final.pdf]

SUPPLEMENTARY MATERIAL

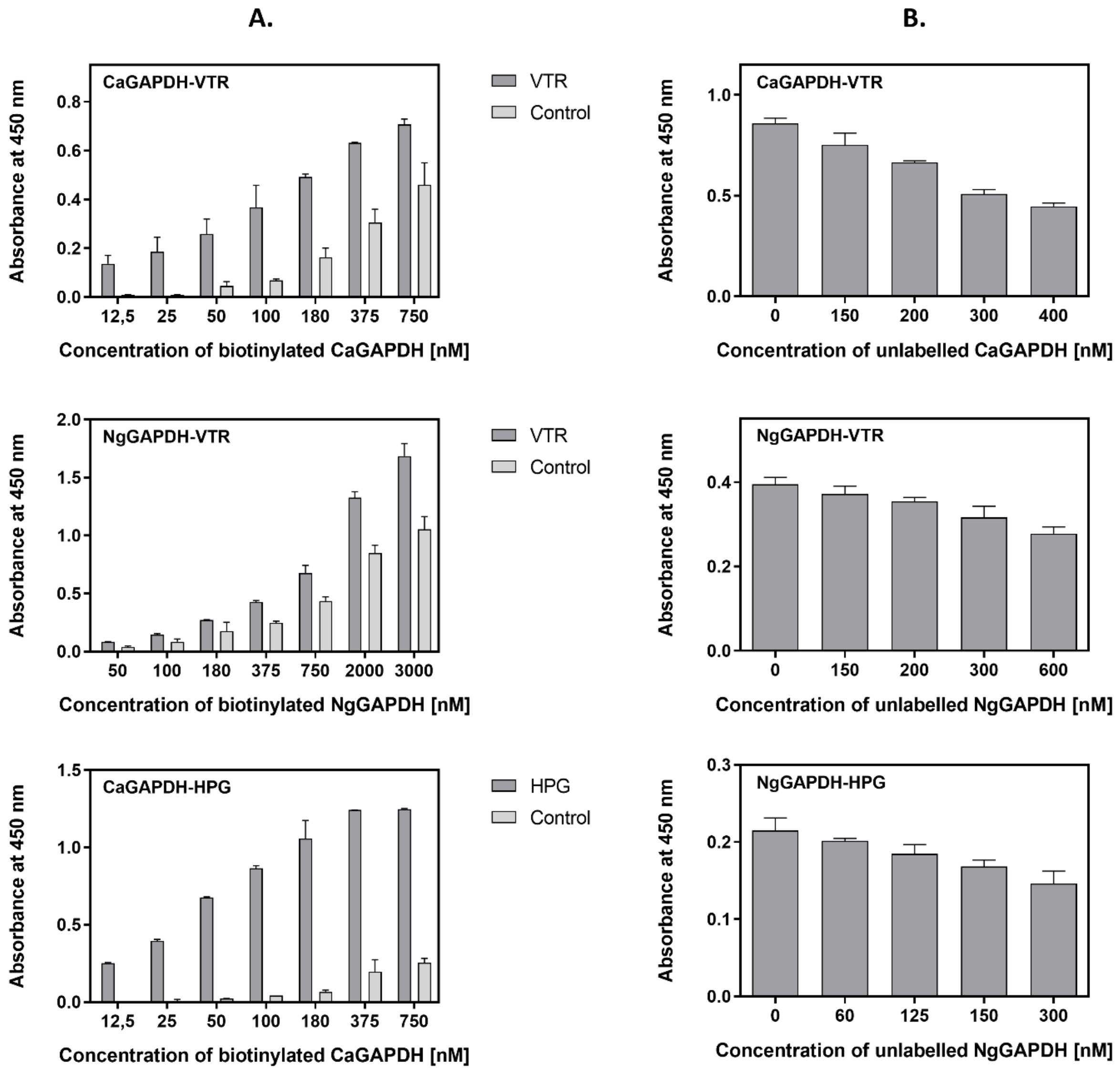

Figure S1. Binding of *C. albicans* or *N. glabratus* GAPDH to human VTR (A) or HPG (B), as analyzed by microplate ligand-binding assay with SA-HRP/TMB detection. Human proteins were immobilized in the microplate wells (3 pmoles per well) by overnight incubation at 4˚C, followed by blocking unoccupied well surface with bovine serum albumin (BSA). (A) Saturable binding assay. Solutions of biotin-labelled GAPDH (50 µl) at increasing concentrations were added to the human protein-coated wells, followed by incubation for 1.5 h at 37 °C. After well washing, the adsorbed biotin-labelled protein was detected with SA-HRP/TMB system. Wells without human protein (only blocked with BSA) served as controls. (B) Competitive binding assay. Mixtures (50 µl) of biotin-labelled GAPDH at a constant concentration of 200 nM for CaGAPDH and 300 nM for NgGAPDH and unlabelled GAPDH at increasing concentrations in a range of 0-600 nM were added to the human protein-coated

wells, followed by incubation for 1.5 h at 37 °C. After well washing, the adsorbed biotin-labelled protein was detected with SA-HRP/TMB system.

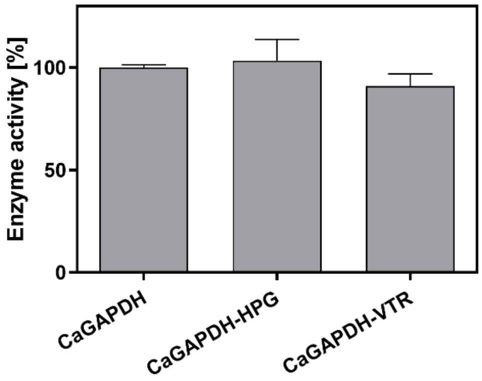

Figure S2. Comparison of the specific enzymatic activity of CaGAPDH after binding human proteins. The proteins were incubated at 37°C for 30 min and the activity was measured with GAPDH Activity Assay Kit (Sigma-Aldrich, cat. no. MAK277). Here 100% is the specific activity of the CaGAPDH protein alone in PBS.

Table S1. List of *S. cerevisiae* strains used in this study.

***S. cerevisiae* strain**	**Description**	**Reference**
UB2155	Empty plasmid	[58]
UB2157	Plasmid with *ALS3*	
UB2158	Plasmid with *EAP1*	
UB2159	Plasmid with *HWP1*	
UB2407	Plasmid with *ALS3*$\Delta_{218-285}$	[88]
UB2416	Plasmid with *ALS3*$\Delta_{325-331}$	
UB2418	Plasmid with *ALS3*$\Delta_{166-225}$	
UB2419	Plasmid with *ALS3*$\Delta_{270-305}$	
UB2420	Plasmid with *ALS3*$\Delta_{277-286}$	
UB2421	Plasmid with *ALS3*$\Delta_{434-830}$	
