# Supplementary figures and images for: Glyceraldehyde 3-Phosphate Dehydrogenase on the Surface of Candida albicans and Nakaseomyces glabratus Cells—A Moonlighting Protein That Binds Human Vitronectin and Plasminogen and Can Adsorb to Pathogenic Fungal Cells via Major Adhesins Als3 and Epa6
Source: Int J Mol Sci. 2024 Jan 13;25(2):1013. doi: 10.3390/ijms25021013 (PMC10815899; doi:10.3390/ijms25021013)

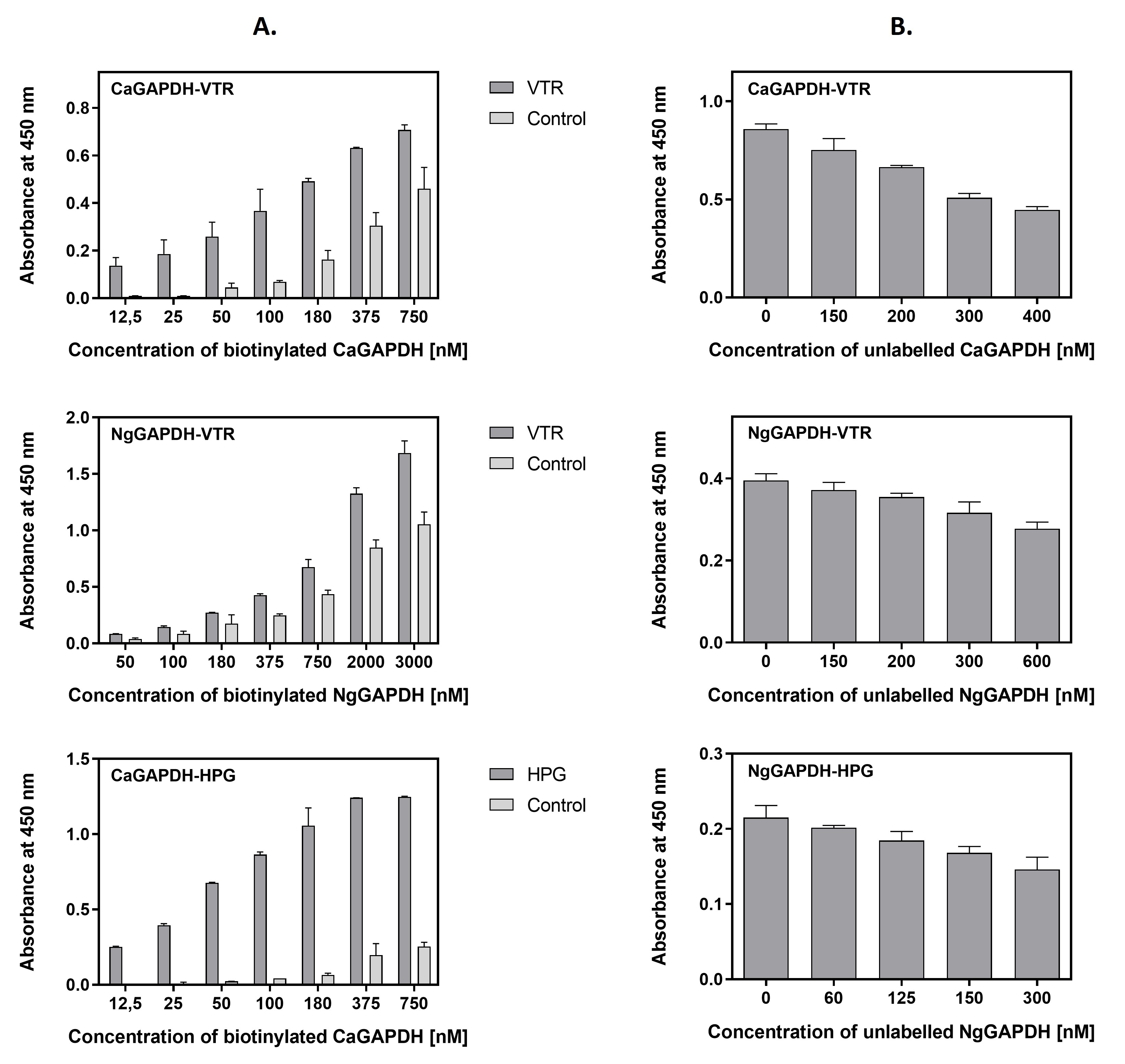

Supplement: Supplementary file 1 [file ijms-25-01013-s001.zip › FigS1.tiff]

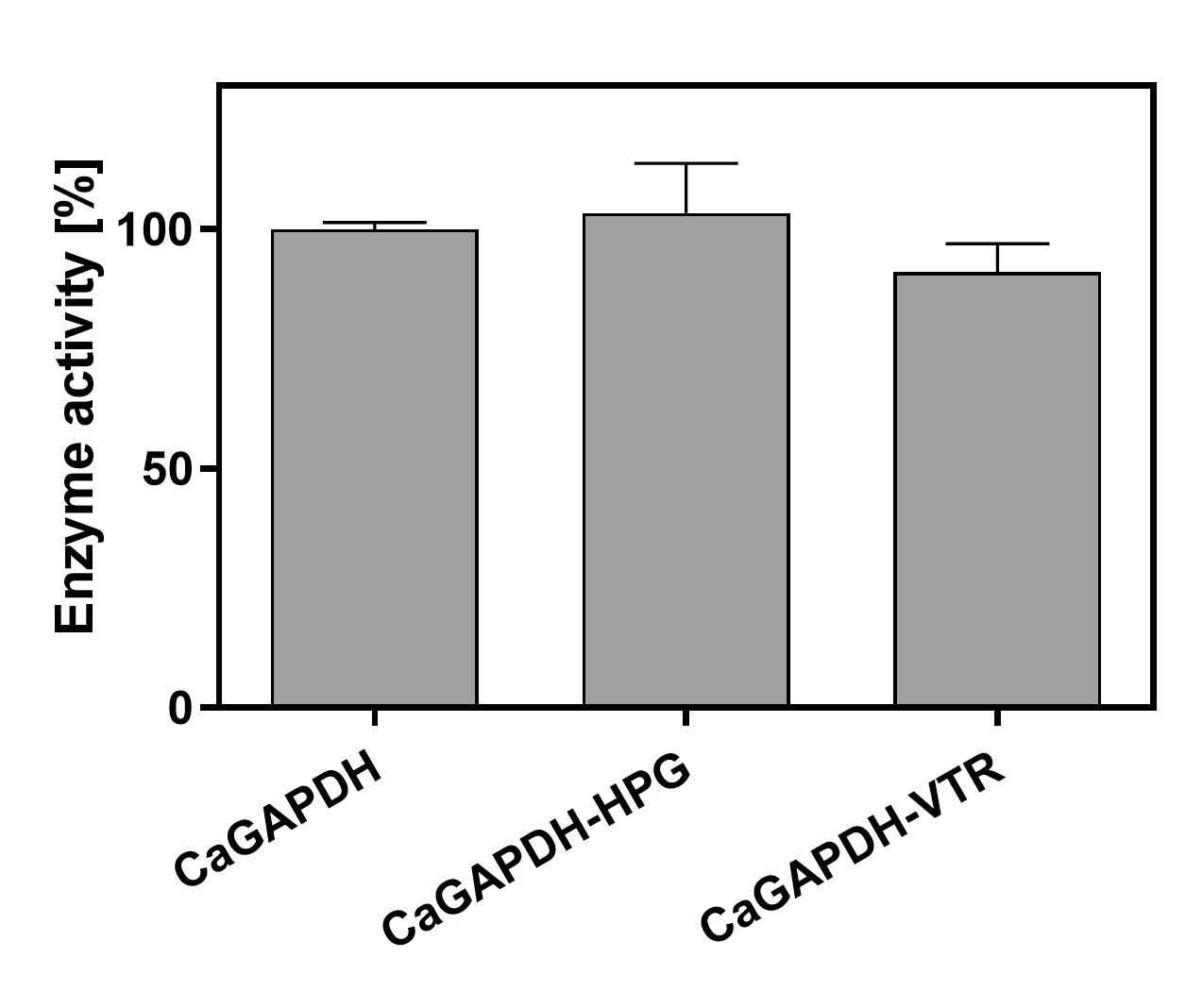

Supplement: Supplementary file 1 [file ijms-25-01013-s001.zip › FigS2.tiff]
